# Study on Parameter Optimization for Support Vector Regression in Solving the Inverse ECG Problem

**DOI:** 10.1155/2013/158056

**Published:** 2013-07-25

**Authors:** Mingfeng Jiang, Shanshan Jiang, Lingyan Zhu, Yaming Wang, Wenqing Huang, Heng Zhang

**Affiliations:** ^1^School of Information Science and Technology, Zhejiang Sci-Tech University, Hangzhou 310018, China; ^2^The Dongfang College, Zhejiang University of Finance and Economics, Hangzhou 310018, China

## Abstract

The typical inverse ECG problem is to noninvasively reconstruct the transmembrane potentials (TMPs) from body surface potentials (BSPs). In the study, the inverse ECG problem can be treated as a regression problem with multi-inputs (body surface potentials) and multi-outputs (transmembrane potentials), which can be solved by the support vector regression (SVR) method. In order to obtain an effective SVR model with optimal regression accuracy and generalization performance, the hyperparameters of SVR must be set carefully. Three different optimization methods, that is, genetic algorithm (GA), differential evolution (DE) algorithm, and particle swarm optimization (PSO), are proposed to determine optimal hyperparameters of the SVR model. In this paper, we attempt to investigate which one is the most effective way in reconstructing the cardiac TMPs from BSPs, and a full comparison of their performances is also provided. The experimental results show that these three optimization methods are well performed in finding the proper parameters of SVR and can yield good generalization performance in solving the inverse ECG problem. Moreover, compared with DE and GA, PSO algorithm is more efficient in parameters optimization and performs better in solving the inverse ECG problem, leading to a more accurate reconstruction of the TMPs.

## 1. Introduction 

The inverse ECG problem is to obtain myocardial transmembrane potential (TMPs) distribution from body surface potentials (BSPs), thus noninvasively imaging the electrophysiological information on the cardiac surface [1, 2]. Generally, approaches to solving this inverse ECG problem can be relied on potential-based model, including epicardial, endocardial, or transmembrane potentials, which is used to evaluate the potential values on the cardiac surface [3] at certain time instants. Moreover, the cardiac electrophysiological information is closely associated with the transmembrane potentials (TMPs) of the myocardial cells. Compared to body surface potentials (BSPs) recordings, TMPs can provide more detailed and complicated electrophysiological information. In this study, we focus on implementing the reconstruction of TMPs from BSPs.

To study inverse ECG problems, various numerical methods have been proposed. In the last decades, regularization methods have been engaged for dealing with the inherent ill-posed property. The regularization techniques include truncated total least squares (TTLS) [4], GMRes [5], and the LSQR [6], which require an appropriate selection of regularization parameters so as to relax the ill-posedness of the inverse ECG problem and to produce a well-posed problem. However, the robustness and the quality of the inverse solution are not always guaranteed; despite that they can more or less deal with the geometry and measurement noises for the inverse ECG problems. In addition, during the solution procedure, the inverse ECG problem can be treated as a regression problem with multi-inputs (BSPs) and multi-outputs (TMPs). Therefore, an alternative method, support vector regression (SVR) method [7], was proposed to solve the inverse ECG problem. Compared with conventional regularization methods, SVR method can produce more accurate results in terms of reconstruction of the transmembrane potential distributions on epi- and endocardial surfaces [1, 8]. SVR is an extension version of support vector machine (SVM) to solve nonlinear regression estimation problems, which aims at minimizing an upper bound of the generalization error instead of the training error [9] by adhering to the principle of structural risk minimization (SRM).

Although SVR is a powerful technique to solve the nonlinear regression problem, it has received less attention in relation to the inverse ECG problems, due to the fact that SVR algorithm is sensitive to users' defined free parameters. The involved hyperparameters of SVR model consist of penalty parameter *C*, insensitive loss function parameter *ε*, and the parameter *σ* for kernel function. Inappropriate parameters in SVR can lead to overfitting or underfitting problems. How to properly set the hyperparameters is a major task, which has a significant impact on the optimal generalization performance and the SVR regression accuracy [10], especially when it comes to predicting the diagnosis of cardiac diseases, because even a slight improvement of prediction accuracy could have a significance impact on the patient's diagnosis [11, 12]. 

In early development stage of the algorithm, a grid search optimizing method [13] and cross-validation method [8] are employed to optimize the hyperparameters. However, these methods are computationally expensive and data intensive. Recently, a number of new algorithms have been proposed for the optimization of the SVR parameters [14, 15]. For example, Wang et al. [16] has proposed a hybrid load forecasting model combining differential evolution (DE) algorithm and support vector regression to deal with the problem of annual load forecasting. In the analysis of predicting tourism demand, the study [17] applies genetic algorithm (GA) to seek the SVR's optimal parameters and then adopts the optimal parameters to construct the SVR models. The experimental results demonstrate that SVR outperforms the other two neural network techniques. Another study [18] tries a new technology, particle swarm optimization (PSO), to automatically determine the parameters of SVR, then applies the hybrid model (PSO-SVR) to grid resource prediction, and the experimental results indicate high predictive accuracy. 

In this paper, the above mentioned optimization algorithms (GA, DE, and PSO) are all adopted to dynamically optimize the hyperparameters of SVR model in solving the inverse ECG problem. For convenience, the SVR model with GA parameter selection is referred to as GA-SVR method, and the other two are termed DE-SVR and PSO-SVR, respectively. In this paper, we attempt to investigate which one is the most effective in reconstructing the cardiac TMPs from BSPs, and a full comparison of the performance for solving the inverse ECG problem will be evaluated.

## 2. Theory and Methodology

### 2.1. Brief Overview of the SVR

In this section, the basic SVR concepts are concisely described; for detailed description, please see [19, 20]. Suppose a given training data of *N* elements {(*x*_*i*_, *y*_*i*_), *i* = 1, 2,…, *N*}, where *x*_*i*_ denotes the *i*th element in *n*-dimensional space; that is, *x*_*i*_ = {*x*_1*i*_,…, *x*_*ni*_} ∈ *R* ^*n*^, and  *y*_*i*_ ∈ *R* is the output value corresponding to *x*_*i*_. According to mathematical notation, the SVR algorithm builds the linear regression function as the following form:

(1) $$\begin{array}{c} f\left(x,\omega \right)=\left(\omega \cdot \varphi \left(x\right)+b\right),\\ \varphi :R^{n}\to F,\omega \in F, \end{array}$$

where *ω* and *b* are the slope and offset coefficients; *φ*(*x*) denotes the high-dimensional feature space, which is nonlinearly mapped from the input space *x*. And the previous regression problem is equivalent to minimizing the following convex optimization problem [see (2)]:

(2) min⁡    12||ω||2subject  to yi−ωφ(xi)−b≤εωφ(xi)+b−yi≤ε.

In this equation, an implicit assumption is that a function *f* essentially approximates all pairs (*x*_*i*_,  *y*_*i*_) with *ε* precision, but sometimes this may not be the case. Therefore, by introducing two additional positive slack variables *ξ*_*i*_ and *ξ*_i_*, the minimization is reformulated as the following constrained optimization problem [shown in (3)]:

(3) min⁡ R(ω,ξ,ξ∗)=12||ω||2+C∑i=1N(ξi+ξi∗)s.t.  yi−(ω,φ(x))−b≤ε+ξi∗    (ω,φ(x))+b−yi≤ε+ξi    ξi,ξi∗≥0, i=1,2,…,N, ε≥0,

where the parameter *C* is the regulator which is determined by the user, and it influences a tradeoff between an approximation error and the weights vector norm ||*ω* | |; *ξ*_*i*_ and *ξ*_i_* are slack variables that represent the distance from actual values to the corresponding boundary values of *ε*-tube.

According to the strategy outlined by Schölkopf and Smola [10], by applying Lagrangian theory and the KKT condition, the constrained optimization problem can be further restated as the following equation:

(4) f(x,ω)=∑i=1N(αi−αi∗)K(xi,x)+bs.t. ∑i=1N(αi−αi∗)=0.

Here *α*_*i*_ and *α*_i_* are the Lagrange multipliers. The term *K*(*x*_*i*_,  *x*) is defined as the kernel function. The nonlinear separable cases could be easily transformed to linear cases by mapping the original variable into a new feature space of high dimension using *K*(*x*_*i*_,  *x*). The radial basis function (RBF) was applied in the study, which has the ability to universally approximate any distribution in the feature space. With an appropriate parameter, RBF usually provides a better prediction performance, so it is adopted in this study as shown in (5):

(5) $$\begin{array}{c} K\left(x_{i},x_{j}\right)=\exp\left(-\frac{{||x_{i}-x_{j}||}^{2}}{2\sigma^{2}}\right), \end{array}$$

where  *x*_*i*_  and  *x*_*i*_  are input vector spaces and *σ* ^2^ is the bandwidth of the kernel function.

In the above equations, there exist three hyper-parameters to be determined in advance, that is, the penalty parameter *C*, insensitive parameter *ε*, and the related kernel function parameters *σ* ^2^. They heavily affect the regression accuracy and computation complexity of SVR. The penalty parameter *C* controls the degree of punishing the samples whose errors go beyond the given value. The insensitive parameter *ε* controls the width of the *ε*-insensitive zone used to fit the training data. The value of *ε* can enhance the generalization capability; with the increase of *ε*, the number of support vectors will decrease, and the algorithmic computation complexity will also reduce. The bandwidth *σ* of the kernel function has a great influence on the performance of learning machine.

In this study, three optimization methods, that is, GA, DE, and PSO, are presented to determine the optimal hyper-parameters of the SVR model. According to [15], the general range of *C*,  *σ* ^2^, and *ε* has been given. In the trial operation, we narrowed it to avoid blindness in the optimization process. In this paper, the set of hyperparameter (*C*, *σ* ^2^, *ε*) is initialized in the given range *C* ∈ [0,10000],  *σ* ^2^ ∈ [0,2], and *ε* ∈ [0,0.0001], where optimization methods (GA, DE, and PSO) are to seek the global optimal solutions. 

In order to calculate the error uniformly, we adopt the same fitness function which plays a critical role in measuring these algorithms performance. The fitness function is determined as follows:

(6) $$\begin{array}{c} \min f=\frac{\sum_{i=1}^{n_{t}}\left(\left|a_{i}-p_{i}\right|/a_{i}\right)}{n_{t}}*100\%, \end{array}$$

where *n*_*t*_ is the number of training data samples, *a*_*i*_ is the actual TMPs of train data, and *p*_*i*_ is the predicted TMPs. The solution with a smaller fitness *f* of the training dataset has a better chance of surviving in the successive generations. The main tool, LIBSVM, was used for training and validating the SVR model [21]. 

### 2.2. Genetic Algorithm (GA) Optimization Method

The GA [22] is a biologically motivated optimization technique guided by the concepts of biological evolution and Darwin's principle of survival of the fittest. It is a computer model of an evolution of a population of artificial individuals. In this study, for the specific optimizing problem of hyperparameters (*C*, *σ* ^2^, *ε*), the process is defined as follows.

**Step 1 [Initialize parameters for GA]**

The population size NP is equal to 20; the dimension of parameter vectors *D* is 3. The termination criterion is set as follows: the number of iterations is set as 30, and the fitness tolerance value is set as 0.001. The probabilities of selection (Stochastic universal sampling), crossover (multipoint crossover) and mutation (mutation operator of the Breeder Genetic Algorithm) that were used herein are 0.9, 0.8, and 0.05, respectively.

**Step 2 (encode chromosomes)**

According to the possible range of parameters *C*, *σ* ^2^, and *ε* given before, the GA utilizes binary encoding method, and each parameter is encoded by 20 bits of binary number. Therefore, the search space is defined as the solution space in which each potential solution is encoded as a distinct chromosome.

**Step 3**

The parameters *C*, *σ* ^2^, and *ε* of each individual are used to build the SVR model. With the BSPs of the training data, the cardiac TMPs are reconstructed. Then the performances of individuals in the generation are evaluated by the specific objective fitness function according to (6). The individual with the minimum fitness value will be selected, and then the chromosome of selected individual is preserved as the best result.

**Step 4**

The basic genetic search operators comprise selection, mutation, and crossover, which are applied orderly to obtain a new generation where the new individual (*C*, *σ* ^2^, *ε*) with the best performance is retained.

**Step 5**

The new best fitness value will be compared to that of the best result, and then select the better one to update the best result.

**Step 6**

This process will not come to an end until the termination criterion is met, and the best chromosome is presented as the final solution; otherwise, go back to Step 4.

### 2.3. Differential Evolution (DE) Optimization Method

Differential evolution [23] is a population-based and parallel direct search method which is used to approximate the global optimal solution. As is mentioned above, the optimization of hyperparameter (*C*, *σ* ^2^, *ε*) can be transformed into solving the minimization of the fitness function Min: *f*(*x*),  *x* = *x*_*iG*_,  *i*_*G*_ = 1,2,…,  and NP is three-dimensional parameter vectors including (*C*, *σ* ^2^,  and  *ε*). Subsequently, each generation evolves by employing the evolutionary operators involving mutation, crossover, and selection operations to produce a trail vector for each individual vector [24], and the detailed evolutionary strategies can be described as follows.

**Step 1 (initialize DE parameters)**

The population size NP is set as 20 [25], and the dimension of parameter vectors *D* is 3. The termination criterion is set as follows: the number of iterations is set as 30, and the fitness tolerance value is set as 0.001. The mutation factor *F* is selected in [0.5, 1], and the crossover rate CR is selected in [0, 1].

**Step 2 (initialize population)**

Set *G* = 0. Generate an NP*∗D *generation which consists of individuals (*C*, *σ* ^2^,  and  *ε*) with uniform probability distribution random values within the control variable bounds of parameter space.

**Step 3**

With the parameters, train the SVR model, and forecast the training datasets. Then calculate the fitness target of all the individuals in the generation, and record the minimum values of the fitness function and set (*C*, *σ* ^2^,  and *ε*) correspondingly as the *x*_best_.

**Step 4 (mutation operation)**

For each target vector *x*_*i*,*G*_, an associated mutant vector is generated according to

(7) $$\begin{array}{c} v_{i,G+1}=x_{\text{best}}+F\left[\left(x_{r_{1},G}-x_{r_{2},G}\right)+\left(x_{r_{3},G}-x_{r_{4},G}\right)\right]. \end{array}$$

The random indexes  *r*_1_,  *r*_2_,  *r*_3_, and  *r*_4_  have to be distinguished from each other and from the current trial index *i*.

**Step 5 (crossover operation)**

In order to increase the diversity of the perturbed parameter vectors, crossover is introduced by the following formula:

(8) uji,G+1={vji,G+1if  (randb(j)≤CR)  or  j=rnbr(i)xji,G+1if  ( randb (j)>CR)and  j≠rnbr(i),

where *u*_*i*,*G*+1_ = (*u*_1*i*,*G*+1_, *u*_2*i*,*G*+1_, *u*_3*i*,*G*+1_), randb(*j*)∈[0,1] is the *j*th evaluation of a uniform random number generator, and rnbr(*i*) is a randomly chosen integer in the range in [1, 3] to ensure that *u*_*i*,*G*+1_ gets at least one element from *v*_*i*,*G*+1_.

**Step 6 (selection operation)**

After the fitness values of the new generation being calculated, then selecting the new best individual as *u*_best,*G*+1_, the selection operation is performed. To decide whether or not it could replace the *x*_best_, the fitness value of *f*(*u*_best,*G*+1_) is compared to that of *f*(*x*_best_). The operation is given as follows:

(9) $$\begin{array}{c} \;\;x_{\text{best}}^{}=\{\begin{array}{cc} u_{\text{best},G+1}^{} & f\left(u_{\text{best},G+1}^{}\right)\;\leq \;f\left(x_{\text{best}}^{}\right)\\ x_{\text{best}}^{} & f\left({u_{\text{best},G+1}^{}}_{}\right)>f\left(x_{\text{best}}^{}\right). \end{array} \end{array}$$

**Step 7**

Termination condition checking: if max iterations *G* is met or the fitness value of *f*(*x*_best_) is reached within the fitness tolerance value, return the recorded global optimal parameter (*C*,  *σ* ^2^, and *ε*); otherwise, go to Step 4.

### 2.4. Particle Swarm Optimization (PSO) Optimization Method

The Particle swarm optimization (PSO) is a bioinspired stochastic optimization technique which was basically developed through simulating social behaviour of birds and insects that keep living by maintaining swarm actions. A particle is considered as a bird in a swarm consisting of a number of birds, and all particles fly through the searching space by following the current optimum particle to find the final optimum solution of the optimization problem [26].

The detailed experimental procedure for selecting the best hyperparameters (*C*,  *σ* ^2^,   and *ε*) is listed as follows.

**Step 1**

Particle initialization and PSO parameters setting [27]: set the PSO parameters including the scope of *C*,  *σ* ^2^, and *ε*; the number of particles is 20; particle dimension is 3. The termination criterion is set as follows: the number of iterations is set as 30 and the fitness tolerance value as 0.001.

**Step 2**

Randomly generate primal population of random particles (*C*,  *σ* ^2^,   and *ε*) and velocities inside the searching space.

**Step 3**

Construct SVR model with the parameters in each particle, and perform the prediction with the training data. Then evaluate the regression accuracy based on the defined fitness function. Then maintain the record of the best performance with the minimum error as the global best solution.

**Step 4**

Particle manipulations: suppose that *X*_*i*_(*k*) and *V*_*i*_(*k*) are the particle and velocity at step *k*, and they are updated into their new values *X*_*i*_(*k* + 1) and *V*_*i*_(*k* + 1) at step *k* + 1 by applying the two equations below:

(10) Vi(k+1)=ωVi(k)+c1r1(pi(k)−Xi(k))+c2r2(pg(k)−Xi(k)),Xi(k+1)=Xi(k)+Vi(k+1)Δt,

where *V*_*i*_ ∈ [*V*_*i*min⁡_,  *V*_*i*max⁡_],  Δ*t* is the unit time step value, and *ω* is the inertia weight. *p*_*i*_(*k*) and *p*_*g*_(*k*) are the best coordinates of particle number *i* and the whole swarm until step *k*, respectively. Positive constants *c*_1_ and *c*_2_ are learning rates while *r*_1_ and *r*_2_ are random numbers between 0 and 1.

**Step 5**

Train the SVR model using the particles (*C*,  *σ* ^2^, and *ε*) in the new generation and forecast the training datasets. After evaluating the fitness function, pick the new best particle as the local best.

**Step 6**

Compare the local best and global best, and choose the better one to update the global best.

**Step 7**

Stop condition checking: if a termination criterion (the number of iterations) is met or the fitness value of the global best is reached within the extent predefined, return the recorded global best as the best parameter (*C*,  *σ* ^2^,   and *ε*); otherwise, go to Step 4.

### 2.5. Simulation Protocol and Data Set

The SVR model is tested with our previously developed realistic heart-torso model [11, 12]. In this simulation protocol, a normal ventricular excitation is illustrated as an example to calculate the data set for the SVR model. The considered ventricular excitation period from the first breakthrough to the end is 357 ms, and the time step is 1 ms, and thus 358 BSPs *φ*_*B*_ and TMPs *φ*_*m*_ temporal data sets are calculated. Sixty data sets (*φ*_*B*_ and *φ*_*m*_) are chosen at times of 3 ms, 9 ms, 15 ms,…, 357 ms after the first ventricular breakthrough which will be used as testing samples to assess the SVR models prediction capability and robustness. The rest 298 in 358 data sets are employed as the training samples for the training and optimal parameter selection procedures. Moreover, during each ventricular excitation period, 412 potentials on the BSPs and 478 potentials on the TMPs are captured. That is to say, the matrix of BSPs is divided into the training data (298 × 412) and testing data (60 × 412), and the matrix of TMPs is also divided into the training data (298 × 478) and testing data (60 × 478). Each SVR regression model is built by using the mapping relations between BSPs training data (298 × 412) and one TMPs training point (298 × 1). Based on all the training data, 478 different regression models were built by using GA-SVR, DE-SVR, and PSO-SVR, respectively. Using the corresponding SVR model, we can reconstruct the corresponding TMPs (60 × 1) from the BSPs testing data (60 × 412). For all testing samples, we can reconstruct the TMPs from the all 478 regression SVR models. 

## 3. The Proposed System Framework

### 3.1. Overview of the SVR Parameters Optimization Framework

**Figure 1:**
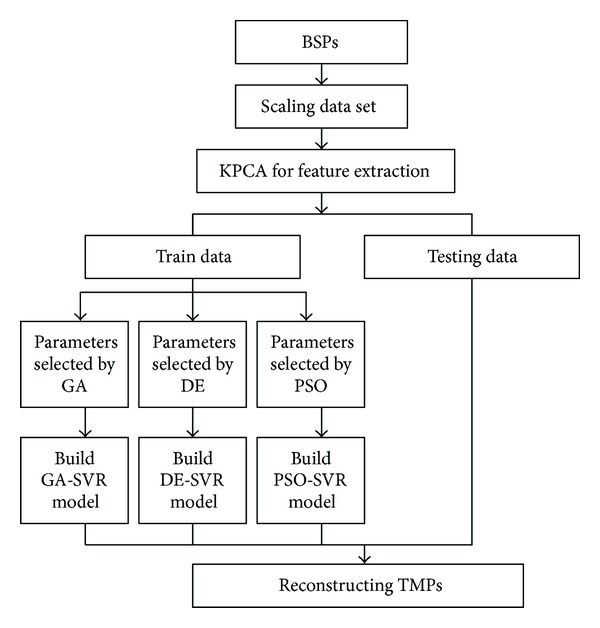
The framework of proposed parameters optimization method for SVR method in solving the inverse ECG problem.

As shown in Figure 1, in the first stage, the original input data is preprocessed by scaling the training data and feature extraction. In the second stage, the hyperparameters (*C*, *σ* ^2^, and *ε*) of SVR method are set carefully by using the GA, DE and PSO optimization methods. Moreover, GA-SVR, DE-SVR, and PSO-SVR are applied to construct the hybrid SVR model, respectively. Finally, according to the effective hybrid SVR models, we can reconstruct the transmembrane potentials (TMPs) from body surface potentials (BSPs) by using these testing samples. 

### 3.2. Preprocessing the Data Set

#### 3.2.1. Scaling the Training Set

During preprocessing stage, each input variable is scaled in order to avoid the potential value from spanning a great numerical range and to prevent numeric difficulties. Generally, all input values including 358 BSPs and TMPs temporal data sets are linearly scaled to the range (0,1) by the following formula [see (11)]:

(11) $$\begin{array}{c} \varphi_{t}^{\prime }=\frac{\varphi_{t}-\varphi_{t\min}}{\varphi_{t\max}-\varphi_{t\min}}, \end{array}$$

where *φ*_*t*_ is the original potential value of each time *t*, *φ*_*t*_′ is the scaled potential value, *φ*_*t*max⁡_ is the maximum potential value of each time *t*, and *φ*_*t*min⁡_ is the minimum potential value of each time *t*. 

### 3.3. Feature Extraction by Using KPCA

The kernel principal component analysis (KPCA) is a nonlinear feature extraction method which is one type of a nonlinear PCA developed by generalizing the kernel method into PCA. Specifically, the KPCA initially maps the original inputs into a high-dimensional feature space *F* using the kernel method and then calculates the PCA in the high-dimensional feature space *F*. The detailed theory of KPCA can be consulted in the paper [28]. 

Feature extraction is a significant task for preprocessing the original input data in developing an effective SVR model. As is investigated in our previous study [12], the SVR with feature extraction by using KPCA has superior performances to that of using PCA or the single SVR method in reconstructing the TMPs. In this paper, the kernel principal component analysis (KPCA) was proposed to implement the feature extraction. And the dimension of BSPs dataset is reduced from 412 to 200 properly, which can reduce the dimensions of the inputs and improve the generalization performances of the SVR method. 

## 4. Results and Discussion

### 4.1. The Result of Parameters Selection

In this study, the GA, DE, and PSO algorithms were proposed to seek the corresponding optimal hyperparameter (*C*,  *σ* ^2^,  and *ε*) of the 478 SVR models.  Table 1 displays the average value and the standard deviations of three parameters in 478 SVR models by using GA, DE, and PSO optimization algorithms. From it, we can find that the gaps among these optimal parameters are obvious by using three different optimization methods. The standard deviations of *ε* are neglected because they are very small.

### 4.2. Comparison of Reconstruction Accuracy

**Figure 2:**
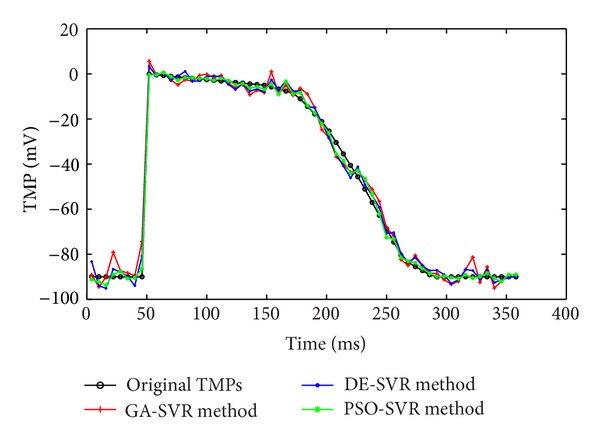
The temporal course of the TMPs for one representative source point on epicardium (50th) over the 60 testing times. The reconstruction TMPs with GA-SVR, DE-SVR, and PSO-SVR methods are all compared with original TMPs.

This study compares the reconstruction performance based on the proposed three intelligent optimization algorithms for selecting the hyperparameters of SVR. By using GA-SVR, DE-SVR, and PSO-SVR hybrid models, two types of experiments for reconstructing the TMPs have been conducted. The first experiment reconstructs the TMPs for one representative source point (the 50th point) on the epicardium over 60 testing times, as is depicted in Figure 2. Since the gaps among these three methods are so slight that we have to add a statistical analysis to validate the results. The mean values and standard deviations of these 60 testing errors between the simulated TMPs and the reconstructed TMPs by using GA-SVR, DE-SVR, and PSO-SVR methods are investigated, as shown in the Table 2. From Figure 2 and Table 2, the PSO-SVR outperforms the GA-SVR and DE-SVR methods in reconstructing the TMPs for one representative source point over all the testing times. Furthermore, the DE-SVR is superior to the GA-SVR method in reconstructing the TMPs. 

**Figure 3:**
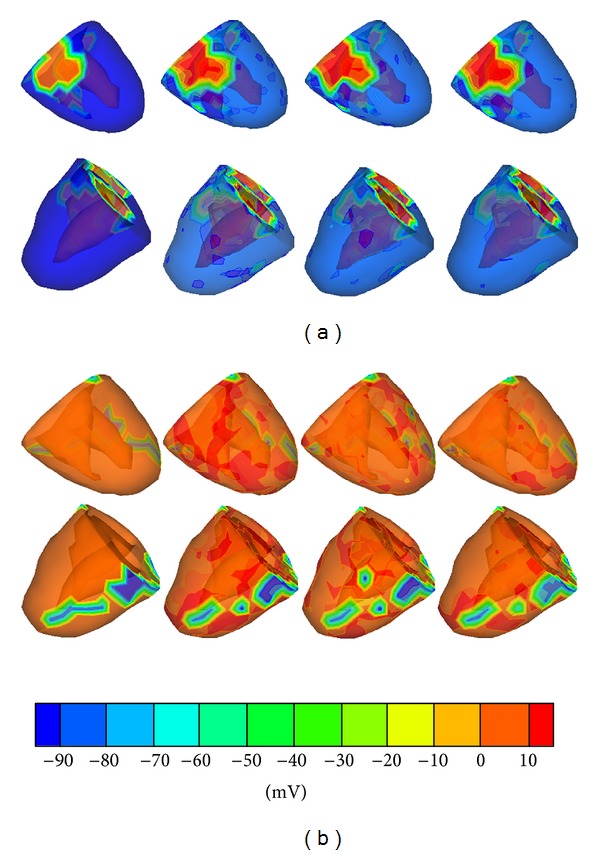
The TMP distribution on the ventricular surface at two sequential testing time points. (a) shows the comparison of reconstructed TMP distributions when the time point is 27 ms and (b) shows the comparison of reconstructed TMP distributions when the time point is 51 ms. Moreover, the upper row shows the TMPs from an anterior view and the lower from a posterior view. In each row, the first figure is the original TMPs, and the rest three are the reconstructed TMPs by using the GA-SVR, DE-SVR, and PSO-SVR methods.

The second experiment reconstructs the TMPs on all the heart surface points at one time instant. These inverse ECG solutions are shown in Figure 3, in which two sequential testing time points (27 and 51 ms after the first ventricular breakthrough) are presented to illustrate the performances of the GA-SVR, DE-SVR, and PSO-SVR methods. It can be seen that, among the three parameters optimization methods, the hybrid PSO-SVR performs better than GA-SVR and DE-SVR methods because its reconstructed solutions are much closer to the simulated TMPs distributions.

Based on the simulation information of TMPs, we can evaluate the accuracy of the reconstructed TMPs of these three different intelligent optimization algorithms at the testing time by mean square error (MSE), the relative error (RE), and the correlation coefficient (CC):

(12) $$\begin{array}{c} \text{MSE}=\frac{1}{n}\sum_{i=1}^{n}{\left(\varphi_{c}-\varphi_{e}\right)}^{2},\\ \text{RE}=\frac{||\varphi_{c}-\varphi_{e}||}{\varphi_{e}},\\ \text{CC}=\frac{\sum_{i=1}^{n}\left[{\left(\varphi_{c}\right)}_{i}-{\bar{\varphi }}_{c}\right]\left[{\left(\varphi_{e}\right)}_{i}-{\bar{\varphi }}_{e}\right]}{||\varphi_{c}-{\bar{\varphi }}_{e}||||\varphi_{e}-{\bar{\varphi }}_{e}||}. \end{array}$$

Here,  *φ*_*e*_  is the simulated TMP distribution at time *t*, and *φ*_*c*_ is the reconstructed TMPs. The quantities ${\bar{\varphi }}_{e}$ and ${\bar{\varphi }}_{c}$ are the mean value of  *φ*_*e*_  and  *φ*_*c*_  over the whole ventricular surface nodes at time *t*. *n* is the number of nodes on the ventricular surface.

**Figure 4:**
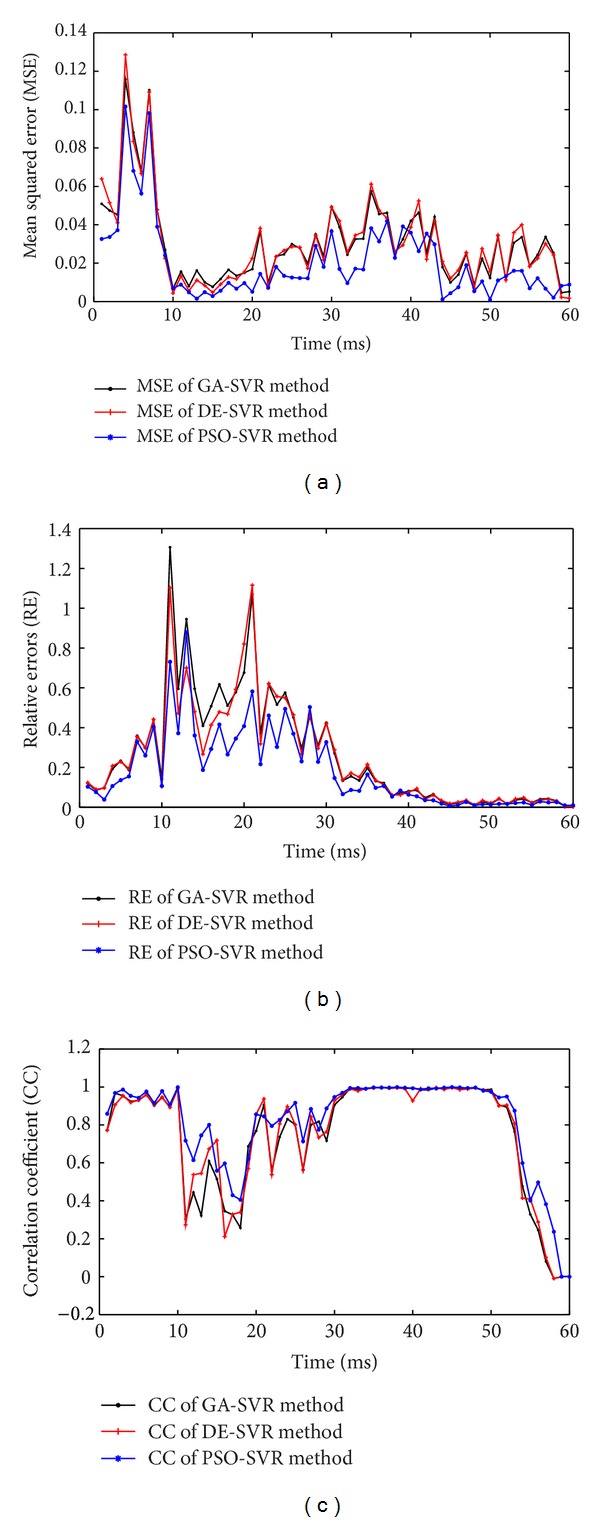
The performances of the reconstructed TMPs over 60 sampling times by using GA-SVR, DE-SVR, and PSO-SVR, respectively. (a) The mean square error (MSE) of the reconstructed TMPs; (b) The relative errors (RE) of the reconstructed TMPs; (c) The correlation coefficient (CC) of the reconstructed TMPs.

**Figure 5:**
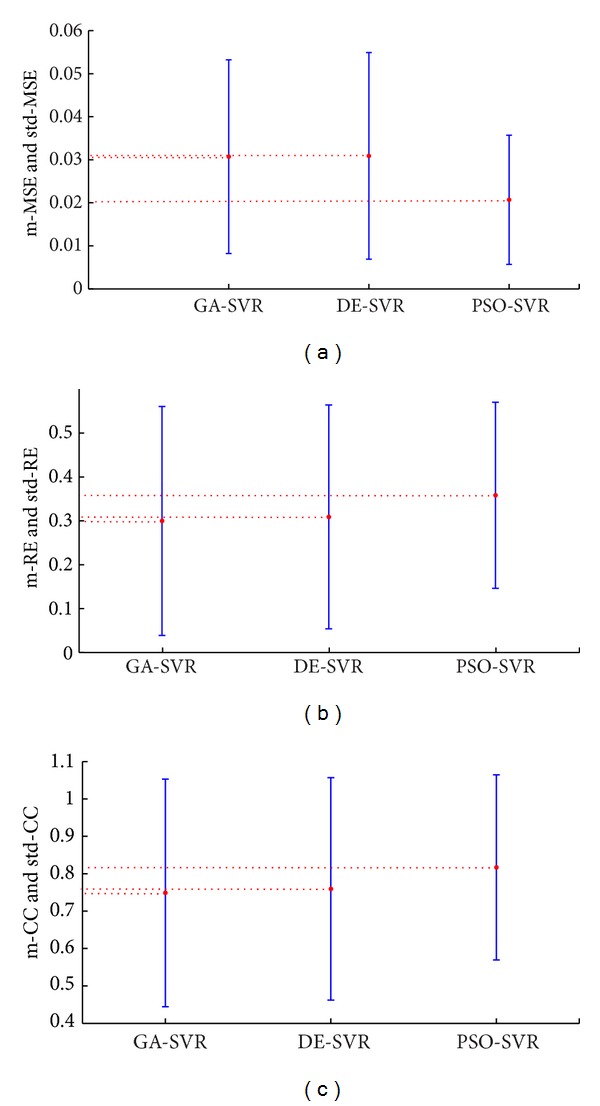
The m-MSE, m-RE, m-CC, std-MSE, std-RE, and std-CC of 60 testing sampling times by using GA-SVR, DE-SVR, and PSO-SVR, respectively, in terms of mean ± standard deviation. (a) m-MSE and std-MSE, (b) m-RE and std-RE, and (c) m-CC and std-CC.

The MSE, RE, and CC of the three sets of reconstructed TMPs over the 60 testing samples can be found in Figure 4. In contrast to GA-SVR and DE-SVR, the PSO-SVR method can yield rather better results with lower RE, MSE, and a higher CC. To validate the robustness of the three methods in reconstructing the TMPs, the quantities m-MSE, m-RE and m-CC, *that is,* the mean value of MSE, RE, and CC at different testing time instants, are presented. Moreover, std-MSE, std-RE and std-CC, *that is*, the standard deviation of MSE, RE, and CC over the whole testing samples, are also investigated. As shown in Figure 5, the mean value of the MSE, RE, and CC over the 60 testing samples obtained by GA-SVR, DESVR, and PSO-SVR methods are presented, and the standard deviations of those 60 MSEs, REs and CCs are also provided.

### 4.3. Comparison of Reconstruction Efficiency

In solving the inverse ECG problem, seeking the optimal parameters in the settled range is time consuming. Therefore, how to successfully excogitate a relatively timesaving optimal method matters significantly in the practical application, particularly in the diagnosis of cardiac disease. Here, we figure out the mean time of the three optimization methods in selecting the optimal hyperparameters of SVR. In order to forecast the 478 TMPs over 60 testing samples on an epi- and endocardial surface, 478 sets of parameters (*C*,  *σ* ^2^, and *ε*) have to be selected in each optimization method and with the testing data, 478 models are built accordingly. In this study, the mean time for selecting the optimal parameters by adopting GA, DE, and PSO is 208.27 s, 342.53 s, and 130.16 s, respectively. It can be seen that PSO can lead to a convergence more quickly and lessen more time than GA and DE. Therefore, according to the comparison of their efficiencies, PSO-SVR method significantly outperforms the GA-SVR and DE-SVR in the reconstruction of the TMP, which is the most efficient one among these three optimization methods.

## 5. Conclusion

In the study of the inverse ECG problem, SVR method is a powerful technique to solve the nonlinear regression problem and can serve as a promising tool for performing the inverse reconstruction of the TMPs. This study introduces three optimization methods (GA, DE, and PSO) to determine the hyperparameters of the SVR model and utilizes these models to reconstruct the TMPs from the remote BSPs. Feature extraction using the KPCA is also adopted to preprocess the original input data. The experimental results demonstrate that PSO-SVR is a relatively effective method in terms of accuracy and efficiency. By comparison, PSO-SVR method is superior to GA-SVR and DE-SVR methods, whose reconstructed TMPs are close to the simulated TMPs. Besides, PSO-SVR is much more efficient in determining the optimal parameters and in building the predicting model. According to these results, the PSO-SVR method can serve as a promising tool to solve the nonlinear regression problem of the inverse ECG problem.

## Figures and Tables

**Table 1:** The average and the standard deviations of optimal hyperparameters (*C*, *σ* ^2^, and *ε*) by using GA, DE, and PSO optimization algorithms.

Parameter	Method		
	GA	DE	PSO
*C*	1447.61 ± 594.32	7624.14 ± 913.67	5.31 ± 3.02
*σ* ^2^	0.64 ± 0.55	1.59 ± 1.38	1.22 ± 1.02
*ε*	1.48*e* − 005	1.80*e* − 005	1.98*e* − 005

**Table 2:** The mean values and standard deviations of these 60 testing errors between the simulated TMPs and the reconstructed TMPs by using GA-SVR, DE-SVR, and PSO-SVR methods at one representative source point (the 50th point on the epicardium).

Indexes	Method		
	GA-SVR	DE-SVR	PSO-SVR
Mean value	2.96	2.69	1.55
Standard deviation	1.92	1.67	1.03

## References

[1] Jiang M, Lv J, Wang C, Xia L, Shou G, Huang W (2011). A hybrid model of maximum margin clustering method and support vector regression for solving the inverse ECG problem. *Computing in Cardiology*.

[2] Ramanathan C, Ghanem RN, Jia P, Ryu K, Rudy Y (2004). Noninvasive electrocardiographic imaging for cardiac electrophysiology and arrhythmia. *Nature Medicine*.

[3] Nielsen BF, Cai X, Lysaker M (2007). On the possibility for computing the transmembrane potential in the heart with a one shot method: an inverse problem. *Mathematical Biosciences*.

[4] Shou G, Xia L, Jiang M, Wei Q, Liu F, Crozier S (2008). Truncated total least squares: a new regularization method for the solution of ECG inverse problems. *IEEE Transactions on Biomedical Engineering*.

[5] Ramanathan C, Jia P, Ghanem R, Calvetti D, Rudy Y (2003). Noninvasive electrocardiographic imaging (ECGI): application of the generalized minimal residual (GMRes) method. *Annals of Biomedical Engineering*.

[6] Jiang M, Xia L, Shou G, Tang M (2007). Combination of the LSQR method and a genetic algorithm for solving the electrocardiography inverse problem. *Physics in Medicine and Biology*.

[7] Brown M, Gunn SR, Lewis HG (1999). Support vector machines for optimal classification and spectral unmixing. *Ecological Modelling*.

[8] Ito K, Nakano R Optimizing Support Vector regression hyper-parameters based on cross-validation.

[9] Vapnik VN (1999). An overview of statistical learning theory. *IEEE Transactions on Neural Networks*.

[10] Schölkopf B, Smola AJ (1998). *Learning with kernels [Ph.D. thesis]*.

[11] Jiang M, Xia L, Shou G, Wei Q, Liu F, Crozier S (2009). Effect of cardiac motion on solution of the electrocardiography inverse problem. *IEEE Transactions on Biomedical Engineering*.

[12] Jiang M, Zhu L, Wang Y (2011). Application of kernel principal component analysis and support vector regression for reconstruction of cardiac transmembrane potentials. *Physics in Medicine and Biology*.

[13] Keerthi SS (2002). Efficient tuning of SVM hyperparameters using radius/margin bound and iterative algorithms. *IEEE Transactions on Neural Networks*.

[14] Sartakhti JS, Zangooei MH, Mozafari K (2011). Hepatitis disease diagnosis using a novel hybrid method based on support vector machine and simulated annealing (SVM-SA). *Computer Methods and Programs in Biomedicine*.

[15] Üstün B, Melssen WJ, Oudenhuijzen M, Buydens LMC (2005). Determination of optimal support vector regression parameters by genetic algorithms and simplex optimization. *Analytica Chimica Acta*.

[16] Wang J, Li L, Niu D, Tan Z (2012). An annual load forecasting model based on support vector regression with differential evolution algorithm. *Applied Energy*.

[17] Chen K-Y, Wang C-H (2007). Support vector regression with genetic algorithms in forecasting tourism demand. *Tourism Management*.

[18] Hu G, Hu L, Li H, Li K, Liu W Grid resources prediction with support vector regression and particle swarm optimization.

[19] Cristianini N, Taylor J (2000). *An Introduction to Support Vector Machines*.

[20] Smola AJ, Schölkopf B (2004). A tutorial on support vector regression. *Statistics and Computing*.

[21] Chang CC, Lin CJ LIBSVM: a library for support vector machines. http://www.csie.ntu.edu.tw/~cjlin/libsvm/.

[22] Oliveri G, Massa A (2011). Genetic algorithm (GA)-enhanced almost difference set (ADS)-based approach for array thinning. *IET Microwaves, Antennas and Propagation*.

[23] Storn R, Price K (1997). Differential evolution—a simple and efficient heuristic for global optimization over continuous spaces. *Journal of Global Optimization*.

[24] Varadarajan M, Swarup KS (2008). Network loss minimization with voltage security using differential evolution. *Electric Power Systems Research*.

[25] Zielinski K, Weitkemper P, Laur R, Kammeyer KD (2006). Parameter study for differential evolution using a power allocation problem including interference cancellation. *Evolutionary Computation*.

[26] Poli R, James K, Tim B (2007). Particle swarm optimization. *Swarm Intelligence*.

[27] Zhang L-P, Yu H-J, Hu S-X (2005). Optimal choice of parameters for particle swarm optimization. *Journal of Zhejiang University*.

[28] Rosipal R, Girolami M, Trejo LJ, Cichocki A (2001). Kernel PCA for feature extraction and de-noising in nonlinear regression. *Neural Computing and Applications*.

